# Complications and risk factors following volar locking plate fixation of distal radius fractures: A 2-institution retrospective study

**DOI:** 10.1097/MD.0000000000049794

**Published:** 2026-07-17

**Authors:** Xiong Zhang, Xiaocong Zhou, Weijiang Bai, Yongliang Liu, Lingde Kong, Bing Zhang

**Affiliations:** a Department of Orthopedic Surgery, The First Hospital of Hebei Medical University, Shijiazhuang, Hebei, China; b Department of Hand Surgery, Hebei Medical University Third Hospital, Shijiazhuang, Hebei, China.

**Keywords:** complications, distal radius fracture, risk factors, 2-center study, volar locking plate

## Abstract

Distal radius fractures are among the most common skeletal injuries and are increasingly treated with volar locking plate (VLP) fixation. Despite its widespread use, postoperative complications remain a significant concern. This study aimed to determine the incidence and spectrum of complications following VLP fixation and to identify independent predictors in a 2-institution cohort. Adult patients who underwent VLP fixation for acute distal radius fractures at 2 tertiary centers between January 2022 and June 2024 were retrospectively reviewed. Inclusion criteria involved operation within 3 weeks of injury and at least 12 months of follow-up. Demographic, clinical, fracture-related, perioperative, and surgeon-related variables were collected from electronic medical records. Complications were defined using standardized criteria and classified as major (requiring reoperation/implant removal) or minor (managed conservatively). Risk factors were evaluated using univariate and multivariate logistic regression. Of 1147 screened patients, 924 met eligibility criteria. Complications occurred in 116 patients (12.6%), comprising 145 events, with 3.6% major and 9.0% minor complications. The most common events were wound problems (21.9%), carpal tunnel syndrome (15.1%), and tendon irritation/rupture (12.3%). Independent predictors included chronic obstructive pulmonary disease (odds ratio [OR]: 2.25, 95% confidence interval [CI]: 1.12–5.62), osteoporosis (OR: 2.03, 95% CI: 1.68–2.43), Association for the Study of Internal Fixation type C fractures versus type A (OR: 1.97, 95% CI: 1.48–2.65), lunate facet collapse ≥5 mm (OR: 3.29, 95% CI: 1.26–7.91), and increased intraoperative blood loss (per 10-mL increment, OR: 1.12, 95% CI: 1.04–1.22). Our findings demonstrate that comorbidities, fracture complexity, and intraoperative factors independently increase complication risk. Individualized perioperative care and meticulous surgical planning may reduce morbidity and improve outcomes in high-risk patients.

## 1. Introduction

Distal radius fractures (DRFs) account for approximately 15% to 20% of skeletal injuries encountered in emergency and orthopedic practice, particularly affecting elderly women with osteoporosis and younger patients exposed to high-energy trauma.^[1]^ The incidence continues to rise globally with population aging, contributing to substantial socioeconomic and healthcare burdens through treatment costs, loss of productivity, and prolonged rehabilitation.^[2,3]^ Volar locking plate (VLP) fixation has become the preferred surgical treatment modality, providing immediate stable fixation and early mobilization compared with external fixation or percutaneous pinning.^[4–6]^ Nevertheless, despite its widespread adoption, postoperative complications remain a major concern, including tendon irritation or rupture, nerve injuries, wound infection, loss of reduction, and implant-related issues.^[7,8]^ In the context of value-based healthcare reimbursement models, minimizing complications and implementing effective perioperative risk stratification have emerged as key priorities in contemporary research and clinical practice.

Recent studies have provided more granular insight into the incidence and spectrum of complications after VLP fixation. Large multicenter and institutional series report complication rates ranging from 6% to 30%,^[9–11]^ though rates as high as 60% have been described depending on definitions applied.^[12]^ In a study of 822 patients, Perregaard et al^[10]^ observed an overall complication rate of 12.3%, including 4.8% major complications and 2.7% requiring hardware removal. A Korean multicenter series of 1955 cases similarly identified tendon rupture (≤0.6%), carpal tunnel syndrome (CTS; 1.8%), infection (4.3%), and implant removal (26%) as leading events.^[13]^ Revision surgery is not uncommon, with reports citing reoperation rates of 2% to 34%.^[14–16]^ Risk factors for complications after VLP fixation are multifactorial, involving patient characteristics (e.g., advanced age, osteoporosis),^[9,17]^ injury severity (higher severity grade, high-energy or multiple injuries), surgical technique (e.g., improper screw length and excessive volar plate prominence),^[18,19]^ and systemic comorbidities (e.g., frailty, chronic obstructive pulmonary disease [COPD], obesity, diabetes),^[17,20]^ and surgeon inexperience (e.g., surgeon volume <30).^[21]^ Nevertheless, limitations exist across prior studies: many are single-center with modest sample sizes, heterogeneous definitions of complications, and inconsistent follow-up durations. Moreover, most fail to integrate patient-related, injury-related, and surgeon-related factors into comprehensive predictive models. This leaves a clear gap in understanding the relative contribution of these factors to complication risk across different practice environments.

The present 2-institution retrospective study aimed to evaluate the incidence and spectrum of complications following VLP fixation of DRFs and to identify independent predictors.

## 2. Methods

This was a 2-institution retrospective cohort study of adult patients who underwent open reduction and internal fixation with a VLP for DRFs in 2 university hospitals between January 2022 and June 2024. Ethical approval for this study was obtained from the Ethics Committee of The First Hospital of Hebei Medical University (No. S00688) and the Ethics Committee of Hebei Medical University Third Hospital (**No. 20210011**). The study was conducted in accordance with the Declaration of Helsinki, and the requirement for informed consent was waived due to the retrospective design and use of anonymized data.

Inclusion criteria were as follows: patients aged ≥18 years; acute DRF operatively treated using a VLP within 3 weeks of injury; and availability of complete medical records and a minimum follow-up of 12 months. Exclusion criteria included pathological (metastatic) fractures, old fractures (>21 days after injury), prior surgery on the affected wrist, combined ipsilateral upper-extremity fractures, nonoperative treatment or surgical procedures other than VLP fixation, and incomplete records or loss to follow-up before 12 months.

The diagnosis of DRF was based on standard anteroposterior and lateral radiographs and/or computed tomography according to the clinical and radiographic criteria recommended by the American Academy of Orthopaedic Surgeons guideline.^[22]^ Fractures were classified using the Association for the Study of Internal Fixation/Orthopaedic Trauma Association (AO)/OTA classification system (types A–C) based on preoperative imaging. Quantitative radiographic parameters were also assessed to confirm instability, including radial shortening ≥3 mm, dorsal angulation >10°, or intra-articular step-off or gap ≥2 mm.^[22]^

Clinical data were retrieved from the electronic medical record systems at both hospitals. Baseline variables included age, gender, body mass index (BMI), comorbidities (hypertension, diabetes, COPD, cardiovascular disease, and osteoporosis), smoking status, fracture characteristics (injury side, mechanism, AO/OTA classification, high- vs low-energy trauma, and involvement of the lunate facet), perioperative factors (surgical delay, American Society of Anesthesiologists, anesthesia type, surgical approach, operation time, blood loss, temporary external fixation, and bone graft use), and surgeon experience (surgeon volume: <30 vs ≥30 DRF cases). Lunate facet collapse was defined as ≥5 mm of subsidence on preoperative radiographs/computed tomography, given prior reports associating ≥5 mm with fixation failure and higher postoperative complication risk.^[23,24]^

The primary outcome was the occurrence of any complication within 12 months after VLP fixation. Secondary outcomes included complication subtypes. Postoperative complications were identified through inpatient records, operative notes, and outpatient follow-up visits. Complications were classified according to established criteria.^[25]^ Tendon-related complications included flexor or extensor irritation, tenosynovitis, or rupture; neurologic complications comprised CTS and peripheral nerve injury; infectious complications included superficial or deep surgical site infection; implant-related events encompassed screw loosening, breakage, dorsal penetration, and plate prominence (Soong grade ≥2); fracture-related complications included loss of reduction, malunion, or nonunion; and other complications included complex regional pain syndrome (CRPS) and wound dehiscence. Major complications were defined as events requiring reoperation or implant removal, while minor complications were those managed conservatively.^[7]^

### 2.1. Statistical analysis

Continuous variables were summarized as means with standard deviations or medians with interquartile ranges, while categorical variables were expressed as frequencies and percentages. Group comparisons between patients experiencing any complication and those without were undertaken using parametric (Student’s *t* test) or nonparametric (Mann–Whitney *U* test) methods for continuous variables, and chi-square or Fisher exact tests for categorical variables, depending on data distribution and expected cell counts. Univariate analyses were first performed to identify potential risk factors. All candidate predictors with a univariate *P*-value < .20 are summarized in Supplementary Table 1, Supplemental Digital Content 1. Variables with a *P*-value < .20 were entered into a multivariate binary logistic regression model to determine independent predictors of complications. Odds ratios (ORs) and 95% confidence intervals (CIs) were reported. Model calibration was evaluated using the Hosmer–Lemeshow goodness-of-fit test. Statistical significance was set at *P* < .05, and model explanatory power was quantified using the adjusted Nagelkerke *R*^2^. All analyses were performed using SPSS version 26.0 (IBM Corp., Armonk).

## 3. Results

**Figure 1.**
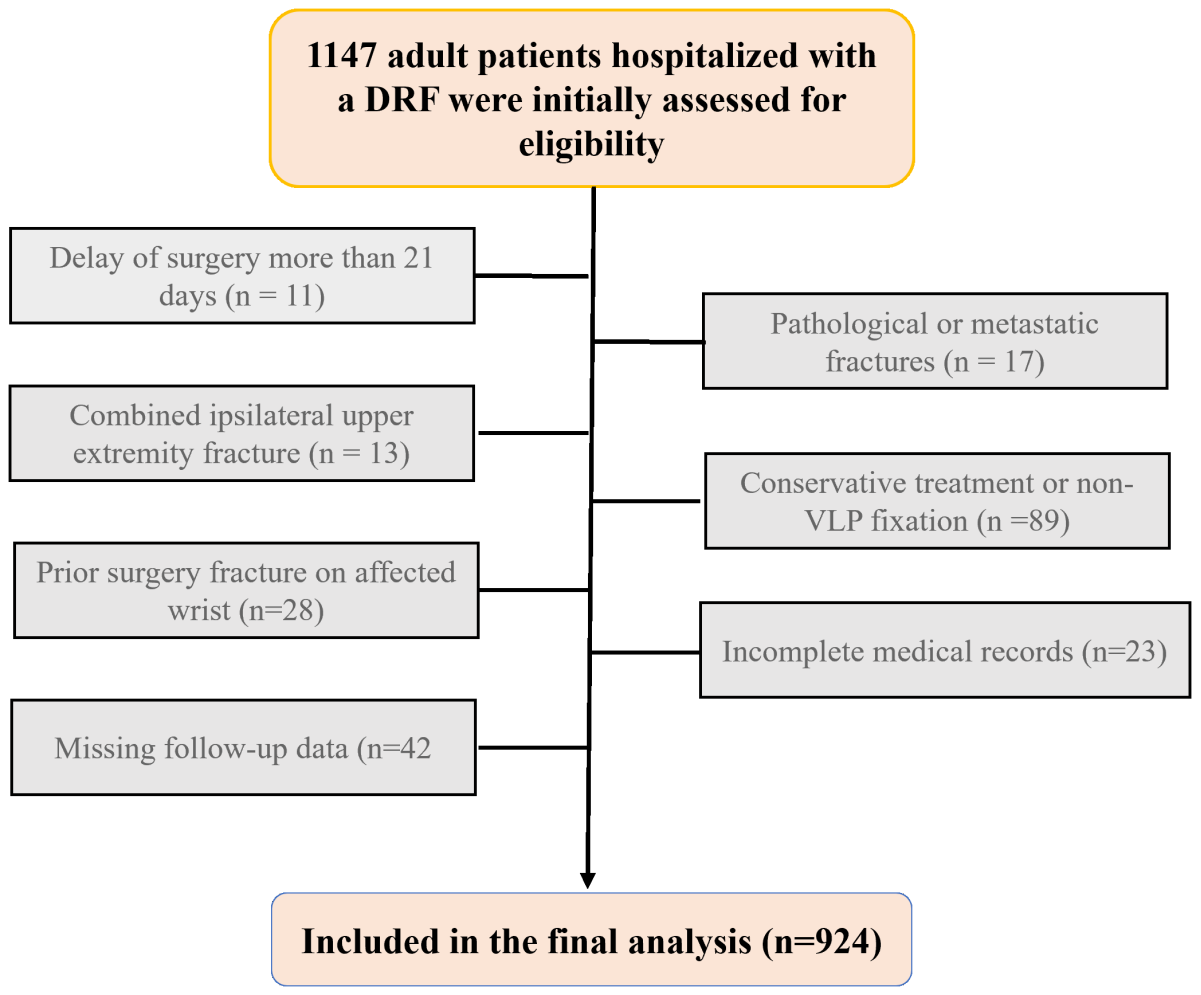
Flowchart for the selection of study subjects. DRF = distal radius fracture, VLP = volar locking plate.

Of 1147 adult inpatients initially assessed, 223 were excluded (delay to surgery >21 days, combined ipsilateral upper-extremity fractures, missing follow-up, non-VLP or conservative treatment, incomplete records, prior ipsilateral wrist surgery, or pathological/metastatic fractures), yielding a final analytic cohort of 924 patients (see flowchart in Fig. 1).

Among the 924 patients included, 116 (12.6%) experienced postoperative complications, accounting for 145 adverse events. Among the 116 patients with complications, 91 (78.4%) experienced a single event, while 25 (21.6%) had multiple concurrent complications (21 with 2, 4 with 3). The most frequent combinations of complications involved wound problems with CTS (n = 8) or tendon irritation (n = 6), followed by CRPS type 2 with CTS (n = 4) and plate/screw-related problems with tendon irritation (n = 4).

Major complications, defined as those requiring reoperation or unplanned implant removal, were observed in 3.6% of patients (n = 33), while 9.0% (n = 83) sustained 1 or more minor complications managed conservatively. The most frequent events were wound problems, including superficial infection (21.9%) and CTS (15.1%), followed by tendon irritation/rupture (12.3%), CRPS type I (11.0%), and implant-related issues (plate/screw penetration, loosening, or breakage; 8.2%; Table 1).

**Table 1:** Complications following VLP fixation of DRF.

Complication	Total events, n (%)	Major events, n (%)	Minor events, n (%)
Wound issues, including superficial infection	27 (18.6)	0	27 (18.6)
Carpal tunnel syndrome	22 (15.2)	4 (2.8)	18 (12.4)
Plate/screw issues (penetration, loosening, breakage)	16 (11.0)	7 (4.8)	9 (6.2)
Tendon irritation/rupture	14 (9.7)	5 (3.4)	9 (6.2)
CRPS type I	12 (8.3)	2 (1.4)	10 (6.9)
Joint stiffness or tendon adhesions requiring surgical release or intensive rehabilitation	10 (6.9)	2 (1.4)	8 (5.5)
Ulnar wrist pain	8 (5.5)	1 (0.7)	7 (4.8)
Radial shortening (≥4 mm)	7 (4.8)	2 (1.4)	5 (3.4)
Distal radioulnar joint instability	6 (4.1)	1 (0.7)	5 (3.4)
Reduction loss	6 (4.1)	3 (2.1)	3 (2.1)
Nerve irritation, paresthesia, or numbness	5 (3.4)	0	5 (3.4)
Deep wound infection	4 (2.8)	1 (0.7)	3 (2.1)
Delayed union	4 (2.8)	1 (0.7)	3 (2.1)
Nonunion	2 (1.4)	2 (1.4)	0
Radial artery injury	2 (1.4)	2 (1.4)	0
Total	145 (100)		

CRPS = complex regional pain syndrome, DRF = distal radius fracture, VLP = volar locking plate.

Between the 2 groups, significant differences were observed in several variables, including COPD (6.9% vs 2.0%, *P* = .002), high-energy trauma (40.5% vs 26.2%, *P* = .001), concomitant osteoporosis (40.5% vs 25.5%, *P* = .001), AO type C fractures (37.9% vs 26.9%, *P* = .035), and initial lunate facet collapse ≥5 mm (10.3% vs 3.3%, *P* < .001). Perioperative variables also differed, with greater intraoperative bleeding (median 140 [110–160] vs 105 [80–135] mL, *P* = .017) and a higher proportion of surgeons with <30 prior DRF cases (18.5% vs 10.9%, *P* = .021) in the complication group. By contrast, no significant differences were found in baseline demographics such as age, sex, BMI, hypertension, diabetes, or cardiovascular disease (Table 2).

**Table 2:** Comparisons between patients with and without documented complications for the data.

Variables	Patients with complications documented (n = 116)	Patients without complications documented (n = 808)	*P*
Gender (male)	68 (58.6)	420 (52.0)	.180
Age (yr)	46.7 ± 15.2	48.1 ± 14.5	.283
BMI	25.7 ± 3.4	25.4 ± 3.2	.803
<24.0	36 (31.0)	298 (36.9)	.186
24.0–27.9	64 (55.2)	437 (54.1)	
≥28	16 (13.8)	73 (9.0)	
Hypertension	28 (24.1)	172 (21.3)	.486
Diabetes mellitus	11 (9.5)	69 (8.5)	.736
Cardiovascular disease	9 (7.8)	46 (5.7)	.379
COPD	8 (6.9)	16 (2.0)	.002
Current smoking	27 (23.3)	179 (22.2)	.786
Injury mechanism			.001
Low-energy	69 (59.5)	596 (73.8)	
High-energy	47 (40.5)	212 (26.2)	
Affected side			.786
Left	61 (52.6)	414 (51.2)	
Right	55 (47.4)	394 (48.8)	
Concurrent osteoporosis	47 (40.5)	206 (25.5)	.001
Dominant side			.569
Yes	64 (55.2)	423 (52.4)	
No	52 (44.8)	385 (47.6)	
Fracture type based on AO classification*			.035
A	52 (44.8)	399 (49.4)	
B	20 (17.2)	192 (23.8)	
C	44 (37.9)	217 (26.9)	
Initial lunate facet collapse (≥5 mm)	12 (10.3)	27 (3.3)	<.001
Time to surgery (d)	3.3 ± 2.4	3.5 ± 2.6	.484
Bone grafting	14 (12.1)	57 (7.1)	.058
Concomitant carpal tunnel release	8 (6.9)	36 (4.5)	.248
Surgeon experience (volume <30 cases)	20 (17.2)	88 (10.9)	.047
ASA score			.266
I–II	94 (81.0)	687 (85.0)	
III–IV	22 (19.0)	121 (15.0)	
Intraoperative blood loss (mL)	140 (110–160)	105 (80–135)	.017
Surgical duration (min)	106 (90–120)	110 (90–130)	.692
Anesthesia (general)			.088
General	17 (14.7)	77 (9.5)	
Regional/local	99 (85.3)	731 (90.5)	
Surgical approach			.918
Flexor carpi radialis approach	106 (91.4)	736 (91.1)	
Henry or modified approach	10 (8.6)	72 (8.9)	
Temporary external fixation	19 (16.4)	67 (8.3)	.005

AO = Association for the Study of Internal Fixation, ASA = American Society of Anesthesiologists, BMI = body mass index, COPD = chronic obstructive pulmonary disease.

* AO/OTA classification: type A = extra-articular fracture; type B = partial articular fracture; type C = complete intra-articular fracture.

In univariate analyses, several variables were associated with higher complication rates, including male gender, higher BMI, COPD, concurrent osteoporosis, high-energy injury mechanism, more severe fracture type, lunate facet collapse, bone grafting, lower surgeon experience, increased intraoperative bleeding, general anesthesia, and temporary external fixation (Supplementary Table 1, Supplemental Digital Content 1, for detailed univariate results). These variables were subsequently entered into the multivariable logistic regression model. In multivariable analysis, COPD (OR: 2.25, 95% CI: 1.12–5.62), concomitant osteoporosis (OR: 2.03, 95% CI: 1.68–2.43), initial lunate facet collapse ≥5 mm (OR: 3.29, 95% CI: 1.26–7.91), AO type C fracture (vs type A; OR: 1.97, 95% CI: 1.48–2.65) and increased intraoperative blood loss (per 10-mL increment, OR: 1.12, 95% CI: 1.04–1.22) remained independent predictors of complications (Table 3). The final multivariate model demonstrated an adequate goodness-of-fit (Hosmer–Lemeshow test, *P* = .411) with an adjusted Nagelkerke *R*^2^ of 0.384, indicating moderate explanatory power.

**Table 3:** Multivariate results for risk factors associated with complications following VLP fixation of DRFs.*

Variables	OR	95% CI		*P*
		Lower limit	Upper limit	
COPD	2.25	1.12	5.62	.038
Concomitant osteoporosis	2.03	1.68	2.43	.001
AO classification†				
A	Reference			
B	0.92	0.71	1.14	.861
C	1.97	1.48	2.65	.012
Lunate facet collapse (≥5 mm)	3.29	1.26	7.91	.003
Intraoperative blood loss (per 10-mL increase)	1.12	1.04	1.22	.026

AO = Association for the Study of Internal Fixation, BMI = body mass index, CI = confidence interval, COPD = chronic obstructive pulmonary disease, DRF = distal radius fracture, OR = odds ratio, VLP = volar locking plate.

* Multivariate logistic regression model adjusted for all variables with *P* < .20 in the univariate analyses, including gender, BMI, COPD, concurrent osteoporosis, injury mechanism, fracture type, lunate facet collapse, bone grafting, intraoperative blood loss, anesthesia mode, and temporary external fixation.

† AO/OTA classification: type A = extra-articular fracture; type B = partial articular fracture; type C = complete intra-articular fracture.

## 4. Discussion

In this 2-institution cohort of patients undergoing VLP fixation for DRFs, postoperative complications occurred in 12.6%, with major complications in 3.6% and minor in 9.0%. Multivariable modeling identified 5 independent predictors: COPD, osteoporosis, AO type C fracture, initial lunate facet collapse ≥5 mm, and greater intraoperative blood loss.

The overall complication rate in our cohort (12.6%) was comparable to that reported by Perregaard et al (12.3%),^[10]^ but markedly lower than that in the multicenter study by Lee et al (26.1%).^[13]^ This variation may be explained by differences in patient demographics (mean age, 47.9 vs 60.3 years), fracture complexity (proportion of AO type C fractures, 28.2% vs 48.8%), follow-up duration (12 vs 18.5 months), and the definition of major complications. Specifically, Lee et al^[13]^ included routine implant removal as a complication, resulting in an overall rate of 26.1%, whereas both our study and Perregaard et al^[10]^ only counted events requiring clinical intervention. These differences should be considered when interpreting the comparative rates of complications after VLP fixation.

Elderly individuals represent the primary population affected by DRFs, and comorbidities should therefore be considered a central component of perioperative management. Consistent with and extending previous research,^[26,27]^ the present study demonstrated that osteoporosis and COPD independently predispose to complications after VLP fixation. The mechanisms appear distinct yet complementary: COPD reduces pulmonary reserve and tissue oxygenation while promoting systemic inflammation, thereby impairing wound healing and increasing susceptibility to surgical site infection^[28]^; osteoporosis compromises trabecular architecture, diminishes screw purchase, and prolongs biological healing, predisposing to secondary displacement, stiffness, and other adverse outcomes.^[29]^ While most DRF studies have focused on diabetes or cardiovascular disease, COPD has rarely been evaluated as an independent factor despite broader orthopedic evidence linking it to pulmonary complications, infection, and readmission.^[26,30]^ Similarly, prior work on osteoporosis has largely emphasized radiographic loss of reduction; our findings extend this evidence by showing that osteoporosis increases overall complication risk beyond fixation failure. These observations underscore the importance of preoperative optimization – including pulmonary assessment and prehabilitation for COPD and bone-health interventions such as vitamin D repletion and antiresorptive or anabolic therapy for osteoporosis – integrated into perioperative planning for elderly DRF patients.

Fracture severity emerged as an important determinant of postoperative complications. AO type C fractures, characterized by articular comminution and loss of metaphyseal support, conferred nearly twice the risk of complications relative to AO type A, corroborating previous evidence that the multiplicity of intra-articular fragments and deficient osseous buttress render anatomical reduction and maintenance technically challenging.^[9]^ Among fracture morphologies, lunate facet collapse appears particularly critical. Up to 50% to 63% of redisplacements occur at the lunate facet, and a residual step-off of ≥2 mm is associated with post-traumatic arthritis rates of 78% to 100%,^[31,32]^ severely affecting long-term outcomes. Our results extend this evidence, demonstrating that initial lunate facet collapse ≥5 mm was associated with a threefold increase in complication risk, which may, mechanistically, involve both intrinsic instability of the lunate facet fragment and the limited capacity of volar plates to adequately support the central and dorsal articular regions. These observations underscore the importance of careful preoperative imaging and planning, and they suggest that in selected high-risk cases, adjunctive fixation strategies – such as dorsal or fragment-specific plating, rafting screws, or arthroscopy-assisted reduction – may be warranted to ensure durable stability and improved outcomes.

We also found that each 10-mL increment in intraoperative blood loss was associated with a 12% increase in the odds of complications. Blood loss itself is unlikely to be causal; rather, it more plausibly functions as a proxy for operative complexity, either reflecting inherently demanding fracture patterns or less meticulous hemostatic control, and thereby serves as a surrogate indicator of surgical difficulty.^[33]^ Few DRF studies have quantified this relationship; thus, its independent effect in our model adds a novel, pragmatic predictor. When blood loss trends higher than expected, surgeons should consider escalation steps – temporary spanning external fixation, staged fixation in the polytrauma/frail patient, a lower threshold for senior assistance, and heightened vigilance for tendon/nerve protection and soft-tissue handling.

Temporary external fixation and bone grafting also showed significant associations with complications on univariate analysis, but these relationships disappeared after multivariable adjustment. This pattern is likely explained by confounding by indication and multicollinearity with markers of fracture complexity. In our series, both procedures were mainly used in patients with AO type C fractures, substantial metaphyseal comminution, larger initial lunate facet collapse, and greater intraoperative blood loss, all of which remained independent predictors of complications in the final model. Thus, temporary external fixation and the use of bone grafts may be better interpreted as surrogate indicators of more severe osseous and soft-tissue injury rather than as independent iatrogenic risk factors per se. Moreover, the relatively small number of patients requiring these adjunctive procedures may have limited the power to detect independent effects in the multivariable analysis.

From a practical perspective, targeted perioperative interventions may help mitigate the identified risks. For COPD patients, preoperative pulmonary evaluation, optimization of bronchodilator therapy, and smoking cessation are recommended to enhance oxygenation and wound healing.^[34]^ For osteoporotic individuals, preoperative correction of vitamin D deficiency, calcium supplementation, and pharmacologic bone-strengthening therapy (e.g., bisphosphonates or teriparatide) should be considered as part of perioperative bone-health optimization, which may in turn improve screw purchase and biological healing.^[35,36]^ In AO type C or highly comminuted fractures, adjuvant fixation methods – including dorsal or fragment-specific plating, rafting screws, or arthroscopic-assisted reduction – may be warranted to achieve stable fixation. Additionally, intraoperative signals should inform surgical tactics: excessive blood loss may serve as a real-time indicator of procedural complexity, prompting tailored strategies. Postoperatively, rehabilitation protocols should be individualized, extending immobilization for unstable constructs or poor bone stock, while promoting early controlled motion once fixation stability is confirmed.

The major strengths of this study include a large, 2-center cohort; standardized complication ascertainment; and multivariable adjustment that disentangles patient, injury, and operative factors. However, several limitations warrant consideration. First, the retrospective design introduces inherent risks of selection bias and incomplete data capture. Second, some potentially relevant variables, such as bone mineral density T-scores, socioeconomic status, or detailed rehabilitation adherence, were unavailable. Third, although 2 centers were included, the findings may not be generalizable to all settings, especially where surgical expertise or implant availability differs. Additionally, the reliance on intraoperative blood loss as a predictor, while practical, may be influenced by recording accuracy. Future research should focus on prospective validation of these risk factors, development of predictive scoring systems that integrate both patient- and surgery-related variables, and exploration of targeted interventions such as enhanced recovery pathways, frailty screening, and advanced fixation techniques for osteoporotic bone.

In conclusion, this multicenter retrospective study identified COPD, osteoporosis, AO type C fractures, lunate facet collapse ≥5 mm, and increased intraoperative blood loss as independent risk factors for complications following VLP fixation of DRFs. These findings highlight the necessity of individualized perioperative planning, meticulous surgical execution, and vigilant postoperative monitoring in high-risk patients.

## Acknowledgments

We are grateful to G.L. of the Department of Orthopedics for his kind assistance. We acknowledge the use of *ChatGPT (GPT-5*, *OpenAI*) to assist with refining the language of the manuscript. All content was subsequently reviewed and verified by the authors, who take full responsibility for the accuracy and integrity of the final work.

## Author contributions

**Conceptualization:** Xiaocong Zhou, Bing Zhang.

**Methodology:** Xiaocong Zhou, Weijiang Bai, Lingde Kong.

**Funding acquisition:** Bing Zhang.

**Data curation:** Xiong Zhang, Weijiang Bai.

**Formal analysis:** Xiong Zhang, Weijiang Bai, Yongliang Liu, Lingde Kong.

**Resources:** Lingde Kong.

**Investigation:** Xiaocong Zhou, Weijiang Bai, Yongliang Liu, Lingde Kong.

**Software:** Yongliang Liu, Lingde Kong.

**Supervision:** Yongliang Liu, Bing Zhang.

**Validation:** Yongliang Liu, Bing Zhang.

**Visualization:** Bing Zhang.

**Writing – original draft:** Xiong Zhang.

**Writing – review & editing:** Xiong Zhang, Xiaocong Zhou, Lingde Kong, Bing Zhang.


