# Supplementary material for: Complications and risk factors following volar locking plate fixation of distal radius fractures: A 2-institution retrospective study
Source: Medicine (Baltimore). 2026 Jul 17;105(29):e49794. doi: 10.1097/MD.0000000000049794 (PMC13384547; doi:10.1097/MD.0000000000049794)
Supplement: Supplementary file 1 [file medi-105-e49794-s001.docx]

**Supplementary Table 1** Univariate analysis results of potential risk factors with P < 0.20 associated with complications following VLP fixation of DRFs

| **Variables** | **OR** | **95% CI** | | ***P*** |
| --- | --- | --- | --- | --- |
|  |  | **lower limit** | **upper limit** |  |
| **Gender (male vs female)** | 1.31 | 0.88 | 1.94 | 0.180 |
| **Body mass index (BMI)** |  |  |  | 0.186 |
| < 24.0 | Reference |  |  |  |
| 24.0-27.9 | 1.08 | 0.92 | 1.26 | 0.384 |
| ≥ 28 | 1.56 | 0.99 | 2.47 | 0.066 |
| **COPD** | 3.67 | 1.53 | 8.77 | 0.002 |
| **Concurrent osteoporosis** | 1.99 | 1.33 | 2.98 | 0.001 |
| **High-energy injury mechanism** | 1.92 | 1.28 | 2.86 | 0.001 |
| **Fracture type based on AO classification** |  |  |  | 0.035 |
| A | Reference |  |  |  |
| B | 0.80 | 0.46 | 1.38 | 0.419 |
| C | 1.56 | 1.01 | 2.40 | 0.045 |
| **Lunate facet collapse (≥ 5mm)** | 3.34 | 1.64 | 6.79 | < 0.001 |
| **Bone grafting** | 1.81 | 0.97 | 3.36 | 0.058 |
| **Surgeon experience (volume <** 30 cases) | 1.71 | 1.00 | 2.90 | 0.047 |
| **Intraoperative bleeding (per 10-ml increase)** | 1.11 | 1.03 | 1.27 | 0.029 |
| **General anesthesia mode** | 1.63 | 0.93 | 2.87 | 0.088 |
| **Temporary external fixation** | 2.17 | 1.25 | 3.76 | 0.005 |

**Abbreviation**: VLP, volar locking plate; DRF, distal radius fracture; OR, odd ratio; CI, confidence interval; COPD, chronic obstructive pulmonary disease
